# Linking Soil Properties and Bacterial Communities with Organic Matter Carbon During Vegetation Succession

**DOI:** 10.3390/plants14060937

**Published:** 2025-03-17

**Authors:** Bin Yang, Jie Zhai, Mengjie He, Ruihao Ma, Yusong Li, Hanyu Zhang, Jiachang Guo, Zhenhua Hu, Wenhui Zhang, Jinhua Bai

**Affiliations:** 1 College of Forestry, Shanxi Agriculture University, Taigu 030801, China; yangbin@sxau.edu.cn (B.Y.); z20213779@stu.sxau.edu.cn (J.Z.); 20233946@stu.sxau.edu.cn (M.H.); z20223901@stu.sxau.edu.cn (Y.L.); zhanghanyu@stu.sxau.edu.cn (H.Z.); s20222472@stu.sxau.edu.cn (J.G.); sxndhzh@163.com (Z.H.); 2 Institute of Forest Ecology, Environment and Nature Conservation, Chinese Academy of Forestry, Beijing 100091, China; mariano_caf@126.com; 3 College of Forestry, Northwest A&F University, Yangling 712100, China

**Keywords:** vegetation succession, soil organic matter carbon, bacterial communities, carbon stabilization, Loess Plateau

## Abstract

Land use change driven by vegetation succession significantly enhances soil carbon storage, yet the microbial mechanisms underlying this process remain poorly understood. This study aims to elucidate the mechanistic linkages between bacterial community dynamics and organic matter carbon stabilization across four vegetation succession stages on the Loess Plateau: abandoned farmland (AF), grassland stage (GS), shrub-land stage (SS), and forest stage (FS). We analyzed soil organic matter carbon (SOM_C) fractions, physicochemical properties, and bacterial communities (16S rRNA sequencing), employing structural equation modeling to quantify causal pathways. The results showed that the content of soil total organic matter carbon (TOM_C), labile organic matter carbon (LOM_C), dissolved organic matter carbon (DOM_C), and microbial biomass carbon (MBC) increased progressively with succession, peaking in the FS, with 23.87 g/kg, 4.13 g/kg, 0.33 mg/kg, and 0.14 mg/kg, respectively. Furthermore, vegetation succession also led to heterogeneity in the bacterial community structure. The number of soil bacterial operational taxonomic units (OTUs) for the four succession stages was 9966, 13,463, 14,122, and 10,413, with the shrub-land stage showcasing the highest OTUs. Nine bacterial taxa were strongly correlated with SOM_C stabilization. Affected by soil bacteria, soil physicochemical properties and litter biomass directly influence SOM_C, with the physicochemical pathway (path coefficient: 0.792, *p* < 0.001) having a greater impact on organic matter carbon than the litter pathway (path coefficient: 0.221, *p* < 0.001). This study establishes that vegetation succession enhances SOM_C content not only through increased litter inputs but also by reshaping bacterial communities toward taxa that stabilize carbon via physicochemical interactions.

## 1. Introduction

Vegetation succession through the establishment of forests or grasslands on abandoned farmland (AF) serves as an effective measure for soil organic matter carbon (SOM_C) storage, thereby aiding in the mitigation of CO_2_ emissions [1]. The world has seen a massive abandonment of agricultural land in recent years [2]. It has gained growing recognition as a successful natural treatment for restoring damaged environments [3]. A significant amount of carbon from the atmosphere is captured and stored by plants in the soil during this ecological process, which could assist in slowing down global warming [3]. Moreover, soil bacterial communities control nutrient cycles through the decomposition and mineralization of soil organic matter, which is essential for plant growth and soil carbon cycling during vegetation succession [4]. Thus, understanding the regulation of bacterial community metabolism during natural vegetation succession is crucial [5].

Vegetation succession modifies soil properties and influences the quality and quantity of litter, which can have either positive or negative effects on the amount and distribution of SOM_C [6]. Nonetheless, understanding the dynamics of SOM_C in the context of vegetation succession is challenging due to the varied sensitivities among the SOM_C fractions [7]. Soil organic matter carbon is typically classified into active and stable carbon using physical and chemical methods [8]. Active components, such as labile organic matter carbon (LOM_C), microbial biomass carbon (MBC), and dissolved organic matter carbon (DOM_C), are more reactive and readily mineralized, serving as sensitive indicators reflecting vegetation changes [9]. In contrast, recalcitrant organic matter carbon (ROM_C) includes stable SOM_C components that can persist in the soil for thousands of years, thus significantly affecting terrestrial carbon sinks [8]. The shift from active to stable carbon fractions during succession is a key process to predicting the long-term impact of vegetation succession on SOM_C storage [8]. Despite the recognized importance of SOM_C fractions in carbon sequestration, the mechanisms linking vegetation succession to SOM_C dynamics, particularly the shift from active to stable fractions, are poorly understood [10]. Therefore, a comprehensive understanding of the interplay between vegetation succession and SOM_C fractions is crucial for developing strategies to enhance carbon sequestration and mitigate climate change [11].

Linking bacterial community to SOM_C sequestration has drawn attention during vegetation succession because of the critical role that soil microorganisms play in bio-geochemical cycles [12]. As various plant species establish and evolve, they contribute distinct organic matter carbon inputs, essential for soil bacterial energy and nutrients [13]. In turn, bacteria transform these complex carbon compounds into simpler forms bioavailable to plants through enzymatic reactions and metabolic processes [14]. Concurrently, the activity of soil bacteria also influences the stability of SOM_C [15]. Soil bacteria can produce extracellular enzymes that break down recalcitrant organic compounds, making them more amenable to microbial decomposition [16]. In the early stages of vegetation succession, microorganisms, in particular bacteria, break down organic matter, facilitating the release of large amounts of organic matter carbon [17]. As succession progresses, the bacterial community undergoes a shift towards a composition that includes carbon-stabilizing microorganisms [18]. Recent studies have shown that certain members of Actinobacteria, Proteobacteria, and Firmicutes, particularly within the families *Micromonosporaceae*, *Bradyrhizobiaceae*, and *Bacillaceae*, possess the unique ability to stabilize and store carbon in the soil. These findings highlight the significant role of specific bacterial taxa in soil carbon sequestration [19]. They convert organic matter carbon compounds into stable forms that can be stored in the soil for extended periods, thus making a significant contribution to mitigating climate change [20]. However, there is still no clear explanation for the overall effects generated by these processes.

The Loess Plateau, which has experienced extensive conversion of farmland to forests, is considered one of the areas with the highest potential for carbon storage [21]. Although it is known that local vegetation succession can impact SOM_C, the exact bacterial taxa responsible for its stabilization across succession stages, and whether its accumulation is more driven by direct bacterial activity or indirect effects mediated through soil physicochemical changes, remain unclear [22,23]. Hence, we hypothesize that: (i) vegetation succession will increase the contents of SOM_C and its fractions, possibly due to litter input and microbial involvement; and (ii) soil physicochemical properties will mediate bacterial effects on SOM_C more strongly than direct litter inputs. Our objectives are as follows: (i) to determine the responses of organic matter carbon fractions (TOM_C, LOM_C, DOM_C, and MBC) to vegetation restoration; (ii) to identify the key bacterial taxa driving organic matter carbon fractions; and (iii) to elucidate the bacterial mechanisms by which vegetation succession drives organic matter carbon accumulation.

## 2. Results

### 2.1. Variation in Soil and Litter Properties Under Succession Stage

Soil properties and litter biomass tend to be enhanced during the long-term process of vegetation succession (Table 1). From AF to FS, SMC, TN, TP, litter biomass, and various SOM_C fractions showed significantly greater values (*p* < 0.05), while BD was significantly lower. Specifically, TOM_C, LOM_C, DOM_C, and MBC were 3.36-fold, 4.05-fold, 11.00-fold, and 14.00-fold greater, respectively, reaching 23.87 g/kg, 4.13 g/kg, 0.33 mg/kg, and 0.14 mg/kg. The most significant increases of TOM_C, LOM_C, DOM_C, and MBC were observed in the grassland and forest stages, with values that were 141.55–300.00% greater compared with earlier stages. Notably, MBC, DOM_C, and LOM_C exhibited higher change rates than TOM_C, with DOM_C and MBC being particularly responsive to vegetation succession.

### 2.2. Variation in Bacterial Community Composition Under Succession Stages

**Figure 1:**
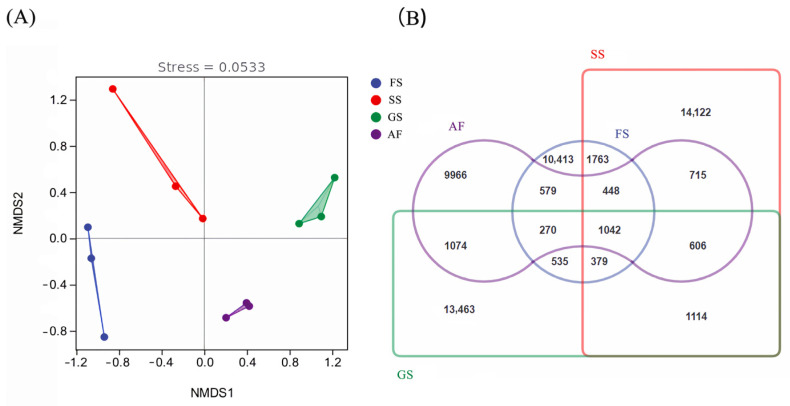
(**A**) Non-metric multidimensional scaling (NMDS) of soil bacterial community composition based on Bray–Curtis distances. (**B**) Venn diagram showing the distribution of soil bacterial OTUs across different vegetation stages. The overlapping sections represent species shared among multiple sample groups, while the non-overlapping sections indicate species unique to each sample group. The numbers indicate the corresponding species counts. AF, abandoned farmland; GS, grassland stage; SS, shrub-land stage; FS, forest stage.

**Figure 2:**
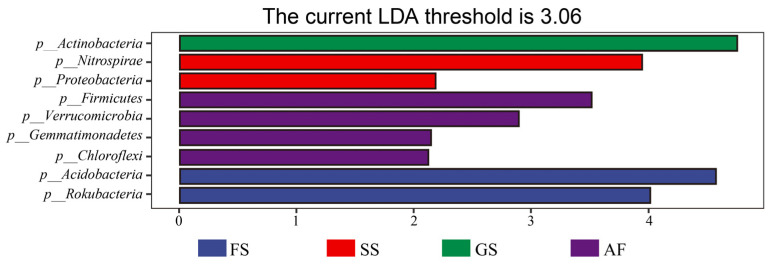
Biomarkers displayed by the least discriminant analysis effect size (LEFSe) taxonomic cladogram (threshold of 2). Samples collected at different succession stages were summarized for LDA. AF, abandoned farmland; GS, grassland stage; SS, shrub-land stage; FS, forest stage.

A total of 25,300 OTUs were obtained through high-throughput sequencing of all soil samples. There were significant differences in bacterial community structure across four succession stages, and most of the OTUs (5232, 8470, 8055, and 5397 for AF, GS, SS, and FS, respectively) were not shared (Figure 1). The lowest value of observed OTUs was registered in soil samples from AF (9966). Interestingly, OTU values did not consistently increase with vegetation succession and reached a maximum at SS (14,122), rather than at the FS (10,413). The LEfSe analysis suggested that nine bacterial phyla (Proteobacteria, Acidobacteria, Actinobacteria, Chloroflexi, Firmicutes, Nitrospirae, Rokubacteria, Gemmatimonadetes, and Verrucomicrobia) dominated the vegetation succession stages (Figure 2). The relative abundance of these dominant bacterial phyla varied among different vegetation types (*p* < 0.05). Additionally, 36 bacterial genera showed significant differences across the vegetation succession stages (Table S1).

### 2.3. Organic Matter Carbon-Associated Soil Bacterial Taxa and Pathways Affecting SOM_C

**Figure 3:**
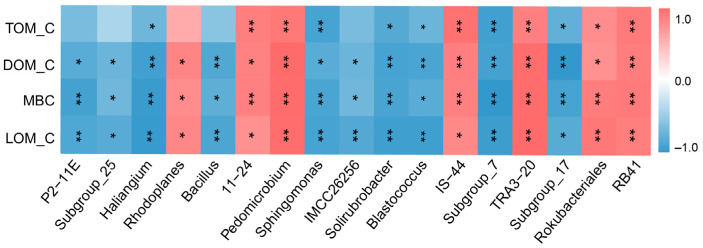
Correlation between soil bacterial and organic matter carbon components. Red and blue indicate positive and negative Spearman correlations, respectively. ** *p* < 0.01, * *p* < 0.05. TOM_C: total organic matter carbon; DOM_C: dissolved organic matter carbon; MBC: microbial biomass carbon; LOM_C: labile organic matter carbon.

The relationship between bacterial communities (top 60 bacterial genera) and SOM_C occurred primarily within six phyla, including Acidobacteria, Rokubacteria, Proteobacteria, Actinobacteria, Firmicutes, and Chloroflexi (Figure 3). Among them, the genera of bacteria related to TOM_C, DOM_C, MBC, and LOM_C were RB41, subgroup_17, *Rokubacteriales*, TRA3-20, subgroup_7, IS-44, *Solirubrobacter*, *Sphingomonas, Pedomicrobium*, 11-24, *Blastococcus*, and *Haliangium*; the genera of bacteria related to DOM_C, MBC, and LOM_C were IMCC26256, *Bacillus*, *Rhodoplanes*, subgroup-25, and P2-11E.

**Figure 4:**
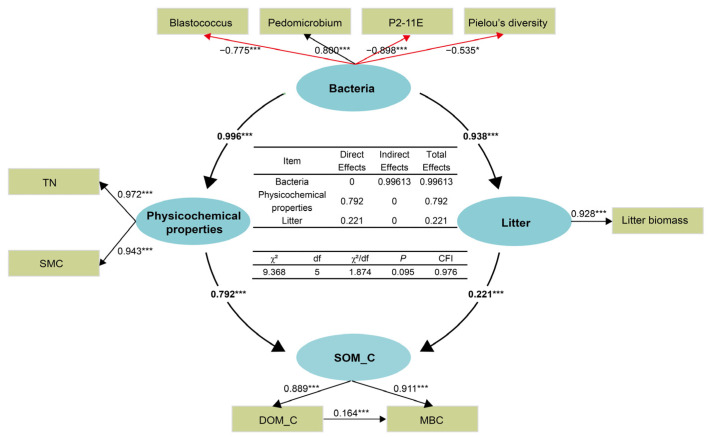
SEM of the effects of soil bacterial on organic matter carbon under vegetation succession. The black arrow indicates a positive correlation and the red arrow indicates a negative correlation. Values associated with arrows indicate standardized path coefficients (* *p* < 0.05, *** *p* < 0.001). SOM_C: soil organic matter carbon; DOM_C: dissolved organic matter carbon; MBC: microbial biomass carbon; SMC: soil water content; TN: total nitrogen; χ^2^: chi-square value; df: degrees of freedom; CFI: comparative fit index.

Structural equation modeling was then performed to build a causality of soil physicochemical factors, carbon cycle-related bacterial genera, and SOM_C (Figure 4). Soil physicochemical properties and litter biomass are the direct indicators that affect SOM_C. Both of them were directly influenced by soil bacteria. Notably, soil physicochemical properties achieved a higher effect (path coefficients of 0.792) through directly influencing the SOM_C (*p* < 0.001) compared with litter (path coefficients of 0.221). Among the taxa associated with SOM_C, the bacterial genera *Blastococcus* (Phylum: Actinobacteria) and P2-11E (Phylum: Proteobacteria), as well as Pielou’s diversity, exhibited a negative correlation with SOM_C.

## 3. Discussion

Soil organic matter carbon content is regulated by soil development related to vegetation succession [24]. The content of SOM_C increased progressively from AF to grasslands, shrub-lands, and forestlands, in agreement with our hypothesis [25]. Abandoned farmland results in significant SOM_C loss due to the lack of sustained inputs of organic matter [26], whereas grasslands and shrub-lands increase vegetation cover and provide relatively abundant root exudates and litter material to increase organic matter carbon [27]. Forest lands feature higher root biomass and litter returns, as well as a better canopy structure, which improves microclimate conditions such as water and heat, providing the optimal environment for SOM_C accumulation [28]. In addition, different organic matter carbon components exhibited varying responses and sensitivities to changes in vegetation succession [29]. In our research, DOM_C was the carbon fraction with the fastest rate of change, followed by MBC, LOM_C, and TOM_C. This was consistent with Meng et al. [30]. The LOM_C/TOM_C can reflect the influence of vegetation types on soil carbon more effectively than the TOM_C content [31]. The smaller the ratio of LOM_C/TOM_C, the more that recalcitrant organic matter carbon (ROM_C) tends to accumulate [9]. In this study, the content of ROM_C (TOM_C minus LOM_C) gradually increased, indicating that the SOM_C tended to stabilize. This process was related to the changes in the amount of litter and the plant root coefficients in the material returned to the soil during vegetation growth [32]. The soil physicochemical properties became relatively stable after long-term vegetation succession, the input and output of organic matter reached a relative balance, and most of the LOM_C transformed into stable mineral-bound organic matter carbon [27]. While our study design does not track vegetation succession along a chronological timeline, we employed the widely accepted “space-for-time substitution” approach, comparing independent sites representing distinct successional stages. This method enables modeling of long-term SOM_C dynamics but inherits limitations from its cross-sectional nature. Nevertheless, our results robustly demonstrate that vegetation succession plays a critical role in driving SOM_C stabilization through preferential accumulation of recalcitrant carbon fractions.

The dynamics of soil bacterial communities during succession facilitate understanding of their development and roles across various successional stages [33]. Contrary to expectations, bacterial OTU richness peaked in the shrub-land stage (14,122 OTUs), not the forest stage. This aligns with intermediate disturbance theory, where shrub-lands balance resource availability (e.g., nutrients and water) and competition, fostering diversity [34]. Late-stage declines reflect niche specialization, as taxa like Actinobacteria outcompete generalists under stable, resource-limited conditions [23]. This finding challenges the assumption that bacterial richness linearly tracks vegetation biomass, emphasizing the role of ecological trade-offs in community succession [34]. This may lead to a shift to less diverse but more resilient and specialized bacterial communities, contributing to the overall stability and function of the ecosystem [34].

In exploring the relationship between vegetation succession and soil bacterial diversity, we found that the bacteria associated with the carbon cycle mainly belong to nine phyla (Figure S1) that were engaged in diverse carbon processes involved in metabolizing and circulating energy within ecosystems [33]. These taxa encode enzymes that are rich in the transport and utilization of carbohydrates, enhancing SOM_C utilization [35]. Eilers et al. [36] found that specific bacterial groups—mainly β-Proteobacteria and γ-Proteobacteria—will preferentially respond to the low-molecular weight carbon compounds. However, Actinobacteria are represented by fast-growing symbionts that are adapted to high-molecular weight carbon [37]. In addition, Chloroflexi, Nitrospirae, Rokubacteria, and Verrucomicrobia were associated to simple carbon compounds (alkyls), whereas Firmicutes and Gemmatimonadetes were more strongly associated with more complex carbon forms (carbonyls) [38]. As vegetation succession reaches the climax stage, the high-molecular weight and complex organic matter carbon soil environment eliminates non-adaptive bacterial communities, reducing bacterial diversity [39].

Soil organic matter carbon is regulated through vegetation succession in two main ways. The first pathway involves the impact of vegetation succession on soil physicochemical properties, which, in turn, influences the bacterial community, ultimately leading to changes in organic matter carbon dynamics. This process can be explained by the fact that vegetation succession increases plant species diversity and canopy density, leading to an increase in soil apoplastic material, which affects plant root systems and nutrient content, thus altering physicochemical properties [40]. Changes in soil bulk density, pH, and N content serve as the primary drivers influencing the composition and diversity of the bacterial community [41]. Soil organic matter carbon content depends on the relative abundance of specific bacterial taxa that mediate nutrient flow from soil to the host plants in exchange for assimilated carbon. As a result, there is a positive correlation between the abundance of bacterial taxa performing such functions and SOM_C content [42].

In our study, we emphasized an additional pathway: soil physicochemical properties, as well as litter biomass, were the most direct indicators that affect SOM_C. Both indicators were directly influenced by soil bacteria. In the fragile ecological environment of the Loess Plateau, bacteria exhibited a more sensitive response to vegetation succession compared with changes in soil physicochemical properties, showing a clear succession trend [43]. Bacterial community holds a crucial role in the degradation of organic matter and the subsequent release of inorganic nitrogen [44]. Plant root exudates and litter provide organic matter for bacteria, promoting nitrogen accumulation [45]. Additionally, bacteria also contribute to soil metabolism, which affects SMC [46]. Soil total nitrogen and SMC, indicators of soil nutrient availability, have substantial influences on SOM_C content [47]. Increased soil moisture creates a favorable environment for the degradation and decomposition of organic matter, thereby increasing the content of LOM_C. Soil total nitrogen is significantly positively correlated with LOM_C and MBC, and higher TN content represents richer nitrogen resources, which is conducive to bacterial growth and activity, ultimately promoting organic matter decomposition and increasing the content of LOM_C and MBC (Figure S2) [48].

In the Loess Plateau ecosystem, vegetation succession directly modulates soil bacterial activity, which in turn alters soil physicochemical properties and litter biomass, creating a feedback loop that further regulates SOM_C. This pathway highlights the heightened sensitivity of soil bacteria to vegetation succession and their pivotal role in organic matter degradation and soil moisture regulation. Key physicochemical indicators, such as TN and SMC, are strongly influenced by bacterial activity and exhibit positive correlations with LOM_C and MBC. These findings underscore the dual role of soil bacteria as both drivers and responders to vegetation succession-mediated changes, emphasizing the critical importance of bacterial-mediated processes in SOM_C stabilization during vegetation restoration.

## 4. Materials and Methods

### 4.1. Study Site

**Figure 5:**
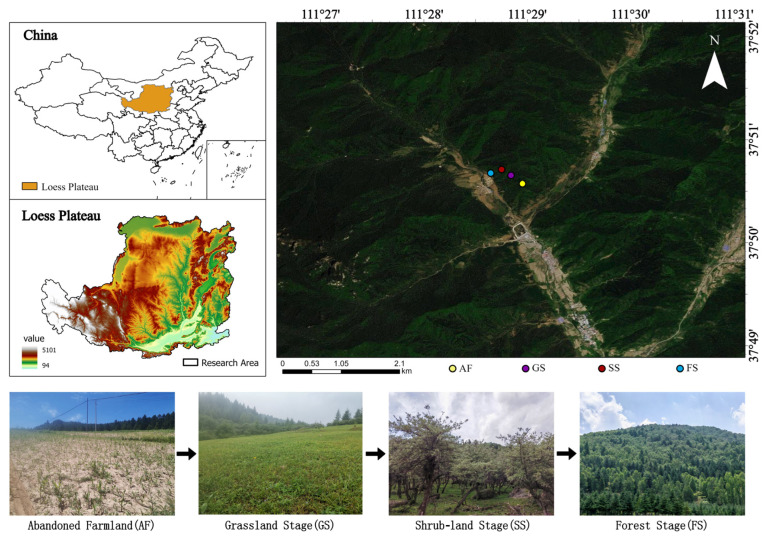
Locations of sampling plots.

This study was conducted in the Xiaowenshan Forest Area (111°24′25″–111°35′25″ E, 37°44′20″–37°53′03″ N, 1345–2659 m ASL), Guandi Mountain, Eastern Loess Plateau, China (Figure 5). This area is a warm-temperate monsoon continental climate zone, with an annual average temperature of 4.3 °C, a frost-free period of 100–125 d, an annual average evaporation of 1268.0 mm, and an annual average rainfall of 822.6 mm, concentrated mainly in June-September [49]. The predominant soil types in the area are Cambisols and Kastanozems. Following farmland abandonment, long-term succession has resulted in the main vegetation patterns consisting of mountain grassland, erect shrubs, and coniferous forests, dominated by *Carex lanceolata* Smith, *Hippophae rhamnoides* L., and *Picea asperata* Franch, respectively.

### 4.2. Experiment Design and Soil Sampling

Throughout the sampling periods, no extreme weather events occurred. A total of 12 sample plots were established in July 2023 across four vegetation succession stages (AF, GS, SS, and FS), with three replicate plots per stage. Within each plot, nine sampling sites were arranged in an S-shape pattern, and soil samples were collected from the 0–20 cm depth at each site after removing surface litter. The nine soil samples from each plot were thoroughly mixed to create a single composite sample per plot, resulting in a total of 12 composite samples (one per plot). Each composite sample was divided into three subsamples for different analyses: (1) soil DNA (stored at −80 °C); (2) DOM_C, MBC and LOM_C (stored at 4 °C); and (3) soil physicochemical properties and TOM_C (stored at room temperature). Bulk density (BD) was determined from a single undisturbed core collected by stainless steel cutting rings (5.0 cm in both diameter and height) at each sampling site. To quantify surface litter biomass, nine 30 cm × 30 cm quadrats were randomly selected within each sample plot. All of the litter in each quadrat was collected, mixed together to form one composite sample, and freshly weighed. The samples were then brought to the laboratory and oven-dried at 65 °C to a constant weight.

### 4.3. Soil Sample Assay

Total organic matter carbon was measured using the potassium dichromate-sulfuric acid oxidation and ferrous sulfate titration [50]. Labile organic matter carbon was determined using the KMnO_4_ oxidation method [51]. Dissolved organic matter carbon was extracted by shaking soil with deionized water for 1 h, followed by filtration through prewashed cellulose acetate filters (0.45 μm pore size) [50]. Microbial biomass carbon was measured by the chloroform fumigation extraction method [52]. Soil water content (SMC) and BD were calculated by the oven-dried method [53]. Total nitrogen (TN) in soils was determined using the Kjeldahl method [53]. Total phosphorus (TP) was determined using the molybdenum-antimony colorimetric method [54].

### 4.4. DNA Extraction, PCR Amplification, and MiSeq Sequencing

Extraction of soil total DNA: A 0.5 g soil sample was taken, respectively, and total soil DNA was extracted according to the operation instructions of the E.Z.N.A. Soil DNA Kit (D5625-01). DNA from each soil sample was extracted for 3 repeats and mixed evenly and stored at −80 °C for subsequent sequencing analysis. High-throughput sequencing and bioinformatics analysis: the V3 to V4 regions of the 16S rRNA gene were amplified using the total DNA of each sample as the template, with primers 338F: 5′-ACTCCTACGGGAGGCAGCA-3′ and 806R: 5′-GGACTACHVGGGTWTCTAAT-3′. Polymerase chain reaction (PCR) system (20 μL): 4 μL of 5×Fast Pfu Buffer, 2 μL of dNTPs (molar concentration 2.5 mmol·L^−1^), 8 μL of forward and reverse primers (molar concentration 5 μmol·L^−1^), Fast Pfu Polymerase, and 10 ng of DNA template. PCR reaction conditions: 95 °C, 5 min; 95 °C, 30 s; 55 °C, 30 s; 72 °C, 30 s, 35 cycles, and 72 °C, 10 min. The amplification products were sequenced by Illumina MiSeq in Shanghai Paisenor Technology Co., Ltd., Shanghai, China. (300 bp paired-end) [55]. The raw sequence data were demultiplexed using the demux plugin, followed by primer trimming with the cutadapt plugin. Sequences were then quality filtered, denoised, merged, and chimeras removed using the DADA2 plugin, followed by Vsearch for clustering. Subsequently, functional gene correction was performed to eliminate non-target fragments, accompanied by a length distribution analysis to identify any irregular sequences. This was followed by species annotation and the construction of a phylogenetic tree to establish genetic distances and relationships between the sequences. Finally, an abundance table of operational taxonomic units (OTUs) was generated, and the data were rarefied to the sequencing depth represented by the lowest 95% of the sample sequences, with a sequencing depth of 60,000 [56]. Additionally, 101,439 optimized sequences were obtained, with an average sequence length of 314 bp. Finally, the complete data were stored in the National Center for Biotechnology Information (NCBI) Sequence Read Archive (SRA) database (PRJNA1111444).

### 4.5. Statistical Analysis

The Shapiro–Wilk test was used to assess the normality assumption for all data, and normalization was applied where necessary. A one-way ANOVA was performed to examine the differences (*p* < 0.05) in SOM_C components and bacterial species at each succession stage using SPSS 21.0 software (IBM, Chicago, IL, USA). Using the “vegan” package in R 4.0.1, we calculated α diversity and conducted a non-metric multidimensional scaling (NMDS) analysis on the variations of soil bacterial communities during the vegetation succession process based on the Bray–Curtis algorithm. Subsequently, linear discriminant analysis effect size (LEfSe) was employed to detect differences in bacterial classifications and functional groups across vegetation succession, using a significance threshold of *p* < 0.05 and log10 LDA > 2. Spearman’s correlation analysis was used to explore the relationships between soil bacteria and organic matter carbon fractions. Structural equation modeling (SEM) was conducted in Amos 18.0 (IBM, Chicago, IL, USA), which was used to analyze the path between microbial communities, SOM_C components, and physicochemical factors, and to verify the hypothesized path of the influence of soil microbial characteristics on soil carbon characteristics.

## 5. Conclusions

This study found that vegetation succession had significant effects on SOM_C content and bacterial community composition. Soil bacteria, physicochemical properties, and litter provided a good explanation for ecosystem carbon sink capacity. In the forest community stage, soil carbon sequestration was the strongest, but its microbial community abundance was not the highest, as it remained at a medium level. Soil bacteria positively affected organic matter carbon by influencing soil physicochemical properties and litter, with soil physicochemical properties having a greater impact than litter. These findings highlighted the importance of soil physicochemical properties in the microbial-driven mechanisms of organic matter carbon sequestration and contributed to a more comprehensive understanding of the intricate relationships between soil physicochemical properties, organic matter carbon, and bacteria in the ecosystem after vegetation restoration.

## Figures and Tables

**Table 1:** Soil and litter properties across different successional stages.

Soil and Litter Properties	Successional Stage (Mean ± SD)				*p*
	AF	GS	SS	FS	
SMC	21.49 ± 0.94 d	24.64 ± 0.84 c	27.87 ± 1.00 b	32.90 ± 0.98 a	<0.01
BD	1.33 ± 0.05 a	1.24 ± 0.05 a	1.22 ± 0.07 ab	1.12 ± 0.06 b	<0.05
TN	1.09 ± 0.03 c	1.10 ± 0.05 c	1.38 ± 0.07 b	1.57 ± 0.10 a	<0.01
TP	0.31 ± 0.01 c	0.32 ± 0.01 bc	0.33 ± 0.01 b	0.36 ± 0.02 a	<0.01
TOM_C	7.10 ± 0.35 c	17.15 ± 0.39 b	17.36 ± 0.79 b	23.87 ± 1.75 a	<0.01
LOM_C	1.02 ± 0.07 d	2.53 ± 0.03 c	2.97 ± 0.12 b	4.13 ± 0.04 a	<0.01
DOM_C	0.03 ± 0.00 d	0.06 ± 0.00 c	0.10 ± 0.01 b	0.33 ± 0.00 a	<0.01
MBC	0.01 ± 0.00 d	0.04 ± 0.002 c	0.07 ± 0.00 b	0.14 ± 0.01 a	<0.01
Litter biomass	0.00 ± 0.00 c	0.00 ± 0.00 c	2.38 ± 0.08 b	3.14 ± 0.09 a	<0.01

SMC, soil water content (%); BD, soil bulk density (g/cm^3^); TN, total nitrogen (g/kg); TP, total phosphorus (g/kg); TOM_C, soil total organic matter carbon (g/kg); LOM_C, labile organic matter carbon (g/kg); DOM_C, dissolved organic matter carbon (mg/kg); MBC, microbial biomass carbon (mg/kg); litter biomass (t/hm^2^); AF, abandoned farmland; GS, grassland stage; SS, shrub-land stage; FS, forest stage; Different lowercase letters indicate a statistical difference among treatments at the 5% level of significance.

## Data Availability

The raw data supporting the conclusions of this article will be made available by the authors on request.

## Supplementary Materials

The following supporting information can be downloaded at: https://www.mdpi.com/article/10.3390/plants14060937/s1, Figure S1: Relationship between soil bacteria, physicochemical properties, and organic matter carbon components; Figure S2. Biomarkers displayed by the least discriminant analysis effect size (LEFSe) taxonomic cladogram (the threshold of 2). Table S1: Variation in the relative abundance of the 60 most dominant bacterial genera under succession stages.

## Abbreviations

The following abbreviations are used in this manuscript:

AF	Abandoned farmland
SMC	Soil water content
BD	Soil bulk density
TN	Total nitrogen
TP	Total phosphorus
GS	Grassland stage
SS	Shrub-land stage
FS	Forest stage
TOM_C	Total organic matter carbon
SOM_C	Soil organic matter carbon
LOM_C	Labile organic matter carbon
ROM_C	Recalcitrant organic matter carbon
DOM_C	Dissolved organic matter carbon
MBC	Microbial biomass carbon
