# Supplementary material for: Linking Soil Properties and Bacterial Communities with Organic Matter Carbon During Vegetation Succession
Source: Plants (Basel). 2025 Mar 17;14(6):937. doi: 10.3390/plants14060937 (PMC11944809; doi:10.3390/plants14060937)
Supplement: Supplementary file 1 [file plants-14-00937-s001.zip › plants-3454848-supplementary.pdf]

Table S1. Variation in the relative abundance of the 60 most dominant bacterial genera under succession stages.

**phylum**	**genus**	***P*** **(<0.05)**
	g_Subgroup_6	0.037
	g_RB41	0.027
	g_Subgroup_25	0.003
	g_11-24	0.041
Acidobacteria	g_Elev-16S-573	0.022
	g_Subgroup_7	0.039
	g_Subgroup_17	0.003
	g_Subgroup_10	0.009
	g_CL500-29_marine_group	0.030
	g_Solirubrobacter	0.001
	g_Aeromicrobium	0.000
	g_Oryzihumus	0.017
Actinobacteria	*g_Mycobacterium*	0.001
	g_67-14	0.007
	g_Blastococcus	0.001
	g_Gaiella	0.026
	g_Acidibacter	0.049
	g_Lysobacter	0.000
	g_mle1-7	0.011
	g_Halomonas	0.002
	g_Rhodoplanes	0.001
Proteobacteria	g_SC-I-84	0.025
	g_Dongia	0.013
	g_Pedosphaeraceae	0.003
	g_bacteriap25	0.000
	g_TRA3-20	0.000
	g_Ellin6067	0.042
	g_P2-11E	0.002
Chloroflexi	g_JG30-KF-CM66	0.000
	g_SBR1031	0.021
Nitrospirae	*g_Nordella*	0.001
	g_Nitrospira	0.000
Firmicutes	*g_Bacillus*	0.005
	g_Lactobacillus	0.049
Gemmaproteobacteria	g_AKAU4049	0.002
Rokubacteria	*g_Rokubacteriales*	0.017

This table lists only the genera of bacteria that are significantly affected. Different lowercase letters indicate significant difference at $p < 0.05$.

Figure S1. Biomarkers displayed by the least discriminant analysis effect size (LEFSe) taxonomic cladogram (the threshold of 2).

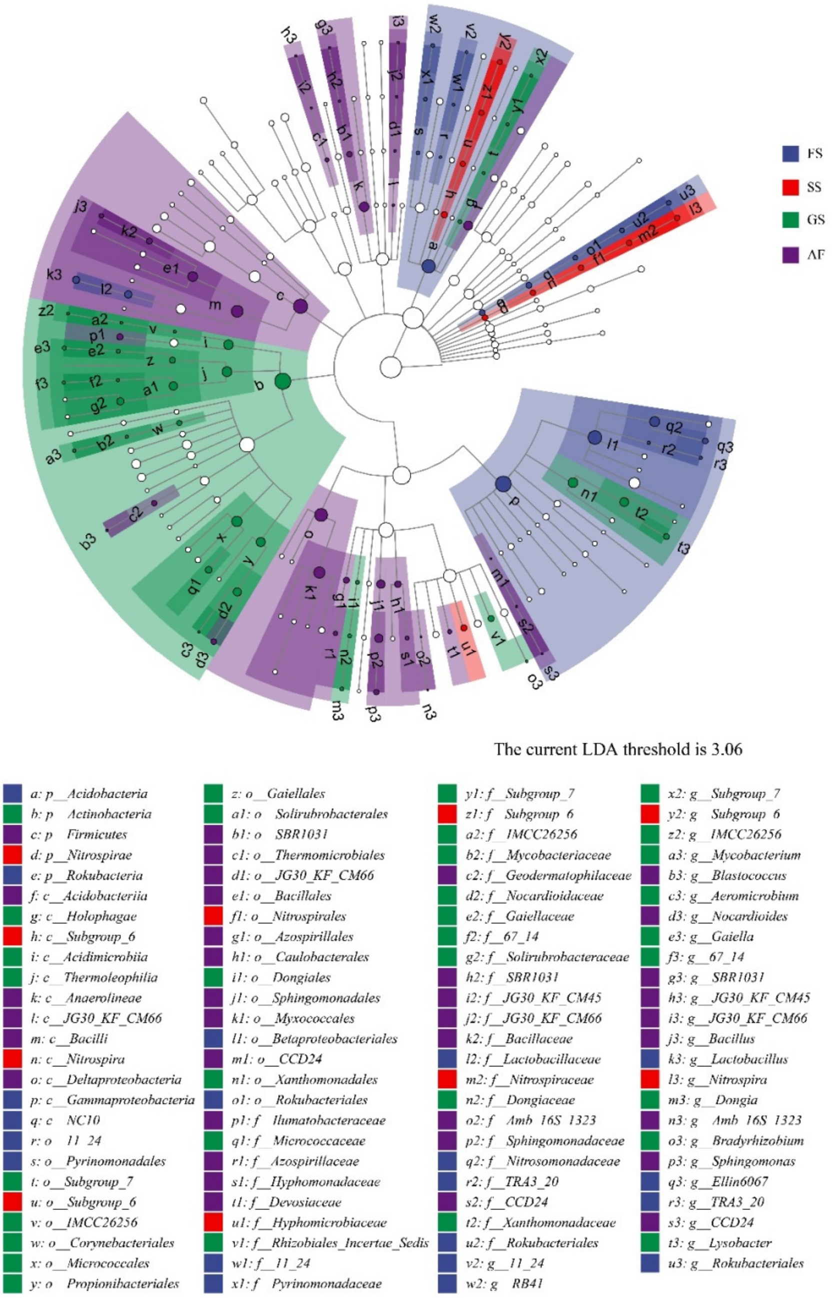

P_, c_, 0_, f_ and g_ in the legend represents the Classification in phylum to genus. Cladogram from the inner ring to the outer ring corresponds to different taxonomic levels of Kingdom, Phylum, Class, Order, Family, Genus and Species, and the line between levels represents the belonging relationship. Node size corresponds to the average relative abundance of the respective taxonomic unit. Hollow nodes represent taxonomic units with insignificant differences between groups, whereas nodes of other colors (such as blue, purple, green, and red) indicate taxonomic units that show significant difference groups and have higher abundances in the respective colored group samples. Letters are used to identify the names of taxonomic units that show significant differences between groups. AF, abandoned farmland; GS, grassland stage; SS, shrub-land stage; FS, forest stage.

Figure S2. Relationship between soil bacteria, physicochemical properties and organic matter carbon components.

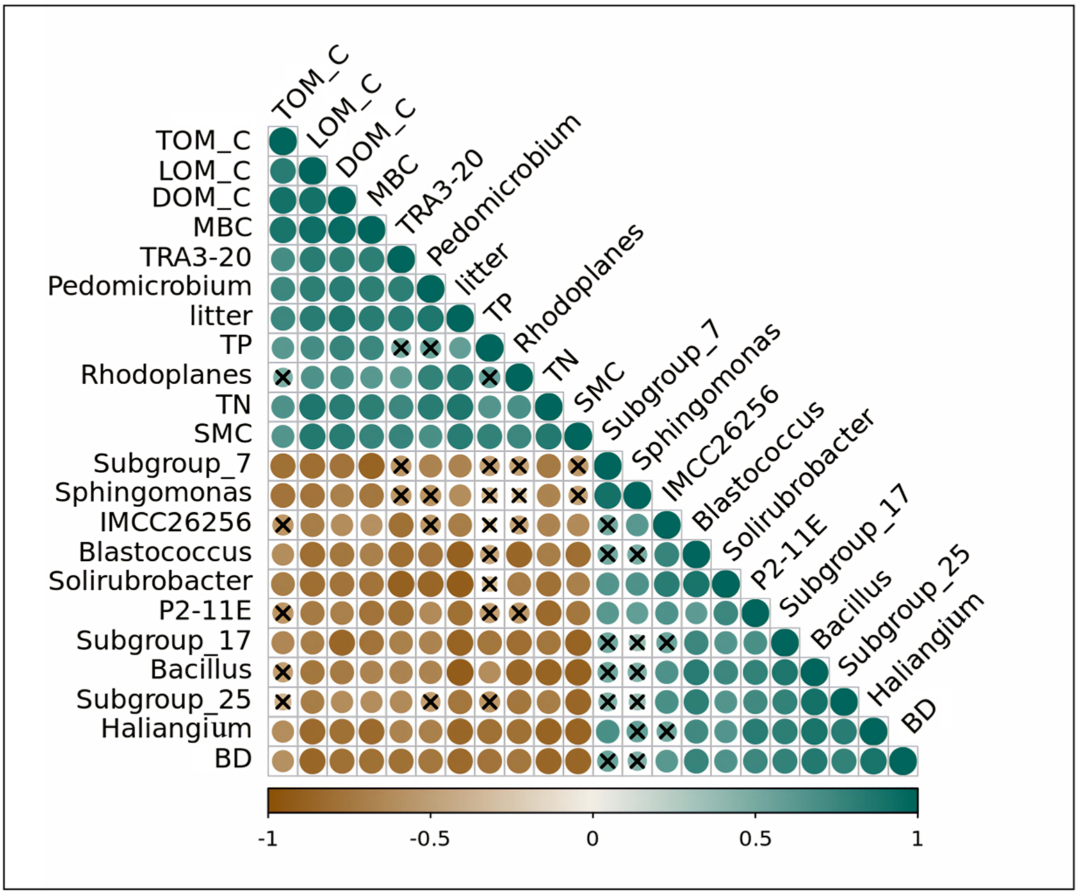

Green indicates a positive correlation, yellow indicates a negative correlation. The darker the color, the stronger the correlation. X represents no correlation. TOM_C: total organic matter carbon, DOM_C: dissolved organic matter carbon, LOM_C: labile organic matter carbon, MBC: microbial biomass carbon, SMC: soil water content, BD: soil bulk density, TN: soil total nitrogen, TP: soil total phosphorus.
